# Production of phytolith and PhytOC and distribution of extractable Si Pools in aerobic rice as influenced by different Si sources

**DOI:** 10.3389/fpls.2023.1146416

**Published:** 2023-08-24

**Authors:** Mohsina Anjum, Nagabovanalli Basavarajappa Prakash

**Affiliations:** Plant Nutrition Laboratory, Department of Soil Science and Agricultural Chemistry, University of Agricultural Sciences, Bangalore, India

**Keywords:** phytolith, PhytOC, rice, Si fertilizers, rice husk biochar

## Abstract

Phytoliths are composed of 66 to 91% SiO_2_ and 1 to 6% organic carbon (C) known as phytolith occluded carbon (PhytOC). PhytOC is critical for long-term C storage in the agroecosystem. A field experiment was carried out to investigate the effect of three different sources of exogenous Si, i.e., diatomaceous earth (DE), silicic acid (SA) and rice husk biochar (RHB) on 1) plant phytolith, C content in phytolith and PhytOC content in different rice organs; 2) relationship between plant phytolith, C content in phytolith, PhytOC content, and soil properties (soil physicochemical properties and readily soluble silicon pools). Different Si sources produced significantly higher phytolith, PhytOC content, and readily soluble Si pools (CCSi, AASi, and ASi) than the control (RDF), with treatment receiving 4 t RHB ha^-1^ outperforming the other treatments. Phytolith and PhytOC production were found to be significantly correlated to soil organic carbon (OC), available nitrogen (N) and potassium (K), 0.01 M CaCl_2_ extractable Si (CCSi) and amorphous Si (ASi) content in the soil. Redundancy analysis showed that treatments receiving 4 t RHB ha^-1^ have a stronger relationship with the CCSi and ASi which majorly contributed to the higher phytolith and PhytOC production. Thus, practices such as Si fertilizers and RHB application have a high potential for phytolith production and PhytOC sequestration, a critical mechanism of the global biogeochemical C sink.

## 1 Introduction

The word phytolith is derived from two Greek words ‘*phyton (φϋτ*o*ν) = plant*’ and ‘*lithos (λίθ*o*ς) = stone*’ meaning ‘plant stone.’ It is a silicified feature of plants, that may accumulate in soils and sediments for several hundreds to thousands of years depending mainly on the morphology and chemical composition of phytoliths and environmental conditions. Rice roots have an active silica uptake mechanism that can absorb up to 90% of the Si in the soil and transport it to the shoots (Mandlik et al., 2020). After translocation, silicic acid is further concentrated and polymerized as amorphous silica (phytolith) passively at concentrations greater than 2 mmol L^-1^ (Mandlik et al., 2020). The concentration of mono silicic acid in the soil does not exceed 2 mmol L^-1^, hence, polymerization occurs only in plants (Mandlik et al., 2020). Phytolith usually takes the shape of the cell where it gets deposited, which means it cannot be mobilized or translocated further (Kumar et al., 2017). Phytoliths are composed of 66 to 91% SiO_2_ with 0.2 to 5.8% organic carbon occluded within them known as phytolith occluded carbon (PhytOC) (Anjum and Prakash, 2021). PhyOC is a recalcitrant source of C (Liu et al., 2023) and can be locked up in the phytolith for up to 1018 years, depending on the morphology, chemical composition and environmental conditions (Tan et al., 2021). Therefore, it is critically important to study different factors influencing phytolith and PhytOC formation.

Rice is a typical Si accumulator plant with up to 10% Si in its above-ground biomass which is higher than the major nutrients (Anjum and Prakash, 2021). The global rice planting area is approximately 167.2 Mha with an annual production of 750 million tonnes (FAO, 2020). India has the largest rice cultivating area accounting for 26.1% (43.79 Mha) of the total area in the world (FAO, 2020). An average rice crop can remove 204-620 kg of Si ha^-1^ (Majumdar and Prakash, 2020a). Rice straw contains 85% of the Si taken up by the plant (Anjum and Prakash, 2021). Continuous removal of rice straw without external supplementation of Si has depleted the plant-available Si (PASi) in the majority of India’s traditional rice fields (Sandhya and Prakash, 2018). Si extracted with CaCl_2_ (CCSi), acetic acid (AASi), and 1% Na_2_CO_3_ (ASi) is considered as PASi proxies as the Si extracted by these reagents is proportional to plant Si uptake. Meunier et al. (2018) also hypothesized that soil phytolith controls PASi. Plant residue decomposition in soil releases phytoliths which are composed of amorphous Si (ASi) particles. Phytoliths are considered the most soluble Si pools compared to other Si minerals (Frysse et al., 2009). Si sources such as Diatomaceous earth and biochar are the sources of ASi (Sandhya and Prakash, 2018; Anjum and Prakash, 2022). Si fertilizers such as diatomaceous earth (Sandhya and Prakash, 2018; Anjum and Prakash, 2022), Foliar silicic acid (ShwethaKumari et al., 2021; Anjum and Prakash, 2022). rice husk biochar (Shetty and Prakash, 2020. Anjum and Prakash, 2022) has been reported to increase PASi, Si uptake and hence crop productivity (Sandhya and Prakash, 2018; Anjum and Prakash, 2022). Increase in productivity is correlated with increases the phytolith and PhytOC production (Anjum and Prakash, 2021).

Different strategies to increase phytolith and PhytOC production has been attempted worldwide. Phytolith and phytOC production has been reported to be correlated with the soil pH and soil nutrient status (Tan et al., 2021). An increase in phytolith and PhytOC production has been reported with the application of nitrogen (N) (Zhao et al., 2016) and nitrogen, phosphorus, and potassium (NPK) fertilizers (Huang et al., 2014) in degraded grasslands and Lei bamboo, respectively. Li et al. (2020a) reported that the application of combined Si-phosphorus (P) increased the plant phytolith concentration and PhytOC content. Combined Si- Nitrogen (N) fertilizers were reported to increase the plant phytolith concentration and PhytOC content in Moso bamboo plantations (Liu et al., 2023). Various Si fertilizers such as basalt powder (Guo et al., 2015), slag-based Si fertilizers (Song et al., 2015; Sun et al., 2019), sodium silicate (Li et al., 2020a) have previously been shown to increase phytolith and PhytOC content in rice in a pot experiment. The results of rice phytoliths and PhytOC differ among different researchers based on the source and type of soil. A field study on the effects of different Si fertilizer sources on rice to select the better source has not been attempted so far. Moreover, the quantitative relationship of PASi and soil physicochemical properties on phytolith or phytOC production has also not been well understood. Therefore, we conducted a field study using three different Si sources, namely diatomaceous earth (DE), silicic acid (SA), and rice husk biochar (RHB). In this study we aimed to (1) investigate the effects of different Si sources on phytolith and PhytOC production in rice; (2) understand the effects of different Si sources on readily soluble Si pools and physicochemical properties of soil and (3) to establish a relationship between phytolith and PhytOC content in plant and soil properties We hypothesized that application of different sources of Si will (1) increase the phytolith and PhytOC production (2) readily soluble Si pools and (3) improve the soil’s physicochemical properties. This work will help towards selecting a practical and cost-effective source of Si for fertilizer management in rice ecosystem.

## 2 Materials and methods

### 2.1 Experimental site and soil

The experiment began in 2015 at C-Block, ZARS, V.C. Farm, Mandya (located at latitude 19° N, longitude 76° E, and 695m altitudes). The soil is classified as Alfisol (Hyperthermic mixed typic *Rhodustalf*). The textural class is sandy loam with 76.54% sand, 6.56% silt, and 16.90% clay. The pH of the experimental soil (2015) was 7.1 (1:2.5 soil-water suspension) and the electrical conductivity (EC) was 0.22 dS m^-1^. At 0-15 cm soil depth, OC content was 11.70 g kg^-1^, available nitrogen (N) was 340.48 kg ha^-1^, available phosphorus (P) was 203.32 kg ha^-1^, available potassium (K) was 348.09 kg ha^-1^, 0.5M acetic acid extractable Si (AASi) concentration was 73.82 mg kg^-1^, 0.01M extractable CaCl_2_ Si (CCSi) concentration was 41.98 mg kg^-1^ and 1% Na_2_CO_3_ extractable amorphous Si (ASi) was 4.91 g kg^-1^.

### 2.2 Cropping system and management

In 2015, the experiment began with the rice-rice cropping system to monitor the inputs, outputs, and the internal silicon transfer in the paddy field to estimate the solute mass balance, status of silicon and bioavailable silicon (Riotte et al., 2018). In 2015, the experiment was started with four replicates of each treatment: (1) barren soil, (2) barren soil with the application of 30 g m^-2^ of diatomaceous earth (DE) from Agri Power Australia Ltd., (3) planted with rice, and (4) rice planted with the application of 30 g m^-2^ of DE before planting. All plots received the recommended dose of fertilizers (RDF) (100:50:50, N: P_2_O_5_: K_2_O kg ha^-1^) as per the package of practices. The entire P (diammonium phosphate, DAP, 18% N and 46% P_2_O_5_) and K (muriate of potash, MOP, 60% K_2_O) dose was applied as basal treatments, while N (urea) was applied in two split doses 30^th^ and 60^th^ day after transplanting. The experiment was repeated in 2016 with rice hull biochar at the rate of 2 t ha^-1^ as the Si source. In 2015 and 2016, KMP-101, locally called Thanu variety, was used for transplanting. However, from July (Rainy season) 2017, the objective of the experiment was changed to assess the effect of different Si sources on rice. The experimental plots were converted to the aerobic rice plot, and the variety grown was changed to BI-33 (Anagha). Three different Si sources were used i.e., diatomaceous earth (DE), concentrated soluble silicic acid (SA), and rice husk biochar (RHB). The composition of DE and RHB is shown in **Table 1**, and concentrated soluble silicic acid (SA), obtained from ReXil Agro BV, Chennai, India, contains 2% Si as soluble H_4_SiO_4_ (ShwethaKumari et al., 2021). As a result, the treatments were modified: (1) T1: Control (RDF alone); (2) T2: RDF + 300 kg DE ha^-1^; (3) T3: RDF + 4 mL SA L^-1^; and (4) T4: RDF + 4 t RHB ha^-1^. The three Si sources RHB, DE and SA were applied at the rate of 4 t ha^-1^, 300 kg ha^-1^ and 4 mL L^-1^, respectively. The entire dose of DE and RHB was applied as a basal before sowing, while the SA was foliar sprayed every 15 days up to reproductive stage of the rice. To avoid the drift to the adjacent plot while spraying, foliar spray was conducted in the early morning with the help of a knapsack sprayer (AGRIMATE, AM 505E) of 20 L capacity. The application rates of DE and RHB fall within the range of field application rates of Si fertilizers (Sandhya and Prakash, 2018) and biochar (Liu et al., 2013), respectively.

**Table 1:** Composition of diatomaceous earth (DE) and rice husk biochar (RHB).

Properties	DE	RHB
pH (1:2.5 water)	9.21	7.39
EC (dsm^-1^ (1:2.5 water))	0.72	1.62
Cation exchange capacity (C mol (p^+^) kg^-1^)	52.00	38.63
Macronutrients (%)		
N	0.03	0.78
p	0.02	0.24
K	0.40	0.96
Si	30.00	31.00
Ca	2.70	0.36
Mg	3.25	0.31
s	0.17	0.05
Micronutrients (Mg kg^-1^)		
Fe	2.00	0.08
Mn	0.02	0.055
B	6.00	8.36
Zn	19.00	63.00
Cu	20.00	31.00
Other elements (Mg kg^-1^)		
Mo	0.10	n.d
Al_2_O_3_	15.30	n.d
Se	1.30	n.d
Cd	0.50	n.d

DE, Diatomite; RHB, Rice hush biochars; n.d, not determine.

### 2.3 Plant sampling and analysis

Plant samples were collected from the field after the crop was harvested in June 2019. The samples were washed with deionized water, dried in an oven at 70°C, and grounded with a ball mill (MM 400, Retsch, GmBH, Germany) for further analysis.

#### 2.3.1 Plant phytolith and PhytOC analysis

**Figure 1:**
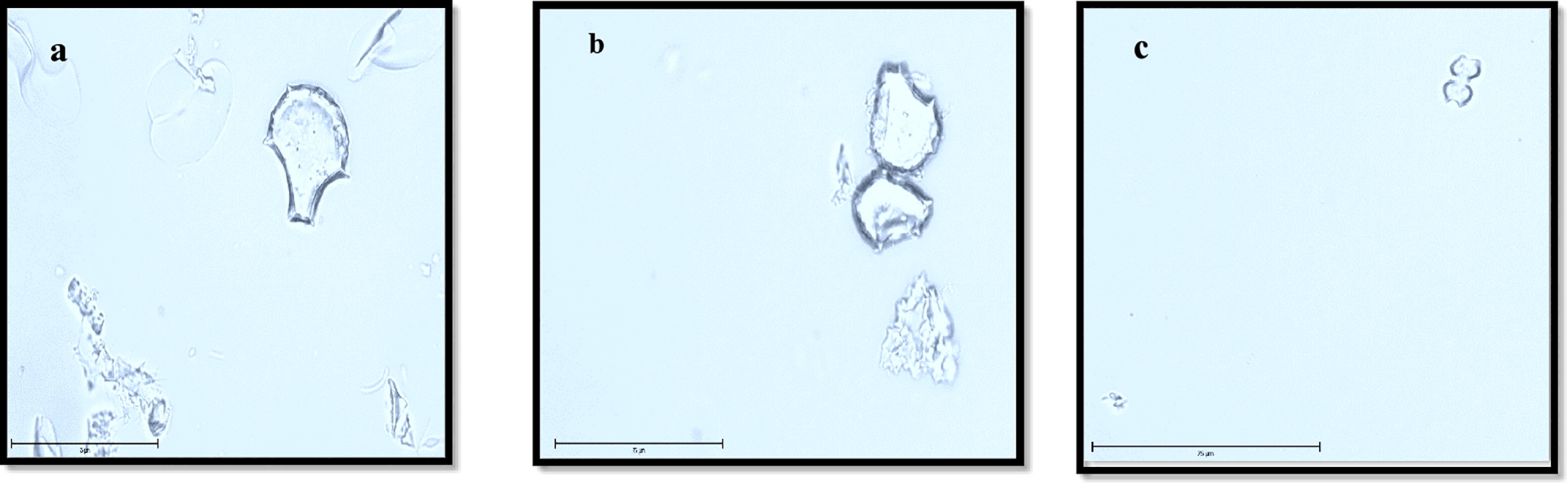
Dominant Phytolith morphotypes identified in rice straw sample under optical microscope (EVOS M700) (400 x or 75 μm) **(A, B)** bulliform, **(C)** bilobate.

Plant phytolith content was extracted using microwave digestion followed by Walkley and Black digest (Anjum and Prakash, 2021). 0.2 g of powdered plant materials were taken into digestion tubes. 3 mL of concentrated HNO_3_, 2 mL of H_2_O_2_ and 0.5 mL of HCl were added to the digestion tube and arranged on the rotor of the microwave digestor (Milestone Start D). The rotor was placed in the microwave and samples were digested for approximately 50 minutes. After 50 minutes samples were rinsed with distilled water and centrifuged four times with a speed of 3500 rpm for 5 minutes. The extracted phytoliths were transferred into pre-weighed centrifuge tubes, dried at 65°C for 72 h in a hot air oven and then weighed. A small amount of phytolith was examined under an optical microscope (EVOS M 7000, Thermo Fisher Scientific, UK) to verify whether organic material external to phytolith were removed extraneous to phytoliths was removed and to identify the morphology of the phytolith (**Figure 1**).

The C content in the isolated phytoliths was determined according to alkali dissolution spectrophotometry (Yang et al., 2014). The procedure is as follows: (a) approximately 0.01 g phytolith was weighed in 10 mL plastic tubes (b) 0.5 mL NaOH (10 mol/L) was added to plastic tubes and allowed to rest for 12 h before being transferred into 30-mL glass tubes (c) 1 mL of K_2_Cr_2_O_7_ solution (0.8 mol L^-1^) and 4.6 mL concentrated sulfuric acid (H_2_SO_4,_ 98% pure) are placed in glass tubes and then heated in a water bath to 98°C for 1 h (d) cooled and distilled water was added to 25 mL of the solution and then it is transferred into 50 mL plastic tubes where tubes are centrifuged at 2500 rpm for 10 min (e) the supernatant is processed by colorimetric analysis at 590 nm, and the absorbance is recorded.

#### 2.3.2 Calculation


C content in phytoliths (%) = (Graph ppm * volume made)/weight of phytolith * 10000

The plant PhytOC content (%) was calculated using the following equation:


PhytOC content of an organ = Phytolith content x C content in phytoliths = 100

### 2.4 Physicochemical analysis of soil sample

After crop harvest, surface soil samples were collected from each plot with a screw auger. Soil samples were air-dried, powdered, and sieved with a 2 mm sieve. The soil pH was measured using a glass electrode with a soil-to-water ratio of 1:2.5 (m: v) (Systronics pH system 362, India). Electrical conductivity (EC in soil water suspension of 1:2.5) was measured with Systronics Conductivity Meter 306, India. The wet oxidation method (Walkley and Black, 1934) was used to analyze the soil OC. The available nitrogen (N) was determined by the alkaline potassium permanganate method (Subbiah and Asija, 1956). Estimation of available phosphorus (P_2_O_5_) by Olsen’s method (Olsen, 1954). Available K was extracted by using 1 N ammonium acetate and was analysed using flame photometry (Jackson, 1973).

### 2.5 Extraction and estimation of readily soluble Si pools

In this study, we extracted three readily soluble Si pools using different extractants: 1) 0.01 M CaCl_2_ (Haysom and Chapman, 1975), 2) 0.5 M acetic acid (Narayanaswamy and Prakash, 2009) and 3) amorphous Si was extracted using 1% Na_2_CO_3_ (Majumdar and Prakash, 2020b). Throughout the paper, Si extracted by 0.01 M CaCl_2_, 0.5 M CH_3_COOH, and 1% Na_2_CO_3_ is referred to as CCSi, AASi and ASi, respectively. The detailed extraction procedures are given below.

#### 2.5.1 0.01 M CaCl_2_ extractable Si

0.01 M CaCl_2_ was used to extract mobile Si fraction i.e., Si present in the soil solution (monomeric silicic acid in most soils). In 50 mL plastic centrifuge tubes, 2.0_ g_ air-dried soil samples along with 20 mL of 0.01 M CaCl_2_ solution were added. Samples were shaken thoroughly in a horizontal shaker for 16 h (Haysom and Chapman, 1975). After that extracted samples were centrifuged at 3000 rpm for 3 minutes and the supernatants were filtered. 0.25 mL reducing agent (1-amino-2-naphthol-4-sulfonic acid - ANSA) was added after 2 minutes. The solution was then left undisturbed for 30 min after the reducing agent was added and the absorbance was measured at 820 nm wavelength using UV visible spectrophotometer (SHIMADZU Pharma spec, UV-1700 series, Japan) with an autosampler (ASC-5). Simultaneously, Si standards (0, 0.5, 1, 2, 3, 4, 5 and 6 mg L^-1^) prepared in the same matrix were analysed by using the UV visible spectrophotometer (Haysom and Chapman, 1975).

#### 2.5.2 0.5 M acetic acid extractable Si

In the present study, 0.05 M acetic acid (CH_3_COOH) was used to extract labile and adsorbed Si (Narayanaswamy and Prakash, 2009). Air-dried soil samples (5.0 g) were thoroughly shaken in an end to end horizontal shaker for 1 hour with 12.5 mL (1:2.5 ratio) of 0.5 M acetic acid (CH_3_COOH) solution in a 50 mL Tarson’s plastic centrifuge tube (Kornodorfer et al., 2001). Extracted samples were centrifuged at 3000 rpm for 3 minutes before being filtered. An aliquot of 0.25 mL was taken from the filtrate and placed in a plastic centrifuge tube with 10.5 mL, 0.25 mL, and 0.5 mL of distilled water, 1:1 hydrochloric acid, and 10% ammonium molybdate solution, respectively. The solution was then left undisturbed for 5 minutes before adding 0.5mL of 20% tartaric acid solution. After 2 minutes, another 0.5 mL of reducing agent (1-amino-2-naphthol-4-sulfonic acid - ANSA) was added. The absorbance was measured at 630 nm after 5 minutes but no later than 30 minutes after the addition of the reducing agent using a UV visible spectrophotometer (SHIMADZU Pharma spec, UV-1700 series, Japan) with an autosampler (ASC-5). Simultaneously, Si standards (0, 0.2, 0.4, 0.8, 1.2 and 1.6 mg L^-1^) prepared in the same matrix were analyzed using a UV visible spectrophotometer.

#### 2.5.3 Amorphous Si

The Na_2_CO_3_ extraction is used for the quantification of amorphous Si (ASi) in soils. This method involves kinetic extraction of the mineral fraction, which has a linear dissolution behavior and nonlinear dissolving ASi fractions (Majumdar and Prakash, 2020b). The principle based on the Na_2_CO_3_ extraction is a weak-base method in which aluminosilicates release Si linearly over time and ASi dissolves at a faster rate than crystalline silicate particles. The ASi content was calculated by determining the linear regression intercept at a constant extraction rate (Majumdar and Prakash, 2020b). In this study, ASi content was determined using time course kinetic extraction with 1% Na_2_CO_3_ (pH 10.8) at 85°C, as described by Majumdar and Prakash (2020b), which involves digestion of the samples for up to 6 h rather than the commonly used 5 h digestion method. 30 mg of dried soil sample was mixed with 40 mL of Na_2_CO_3_ in round bottom polypropylene tubes and placed on the water shaker bath (MEMMERT GmbH + Co. KG, Germany) at 85°C ± 0.6°C with caps slightly loosened to vent gases. Later, at 3, 4, 5 and 6 h, 1 mL of aliquot was taken. Which was neutralized and acidified in a 10 mL Tarson tube with 9 mL of 0.021 N HCl. The molybdenum blue colorimetric method was used to determine the Si concentration in the collected sub-sample using the Silicon kit - SpectroquantR reactant (Merck). A UV visible spectrophotometer was later used to measure the intensity of the blue color at 820 nm.

### 2.6 Calculations and statistical analysis

The data presented in the study is the average of the four replicates. All datasets were analyzed statistically using one-way ANOVA with SPSS and Star software packages at a significance level of P<0.05 via Tukey test. Redundancy analysis (RDA) was carried out to determine the interrelationship between plant phytolith and soil parameters. RDA is an intriguing extension of PCA in which the response variables are explicitly modelled as a function of explanatory variables. In this study, the phytolith and PhytOC parameters were considered as response variables, whereas soil parameters (pH, EC, OC, available N, P, K, CCSi, AASi and ASi) were explanatory variables. To determine the structure and direction of the interdependences among the response variables a PCA was performed using R x 64 4.0.1 software using the ggplot2 package. The correlation matrix was used to extract principal components. Kaiser-Meyer-Olkin (KMO) and Bartlett’s tests were used to determine the suitability of the data for PCA (Maurya et al., 2021). KMO test results greater than 0.50 and Bartlett’s significance level p<0.05, indicated a significant relationship between different variables. The RDA’s graphical output is a triplot, which is made up of two biplots stacked on top of each other. The graph’s three components are 1) explanatory variables, 2) response variables, and 3) treatment. Monte Carlo permutation test with PERMANOVA with 999 permutations is used to determine the order of importance of explanatory variables (adonis function). The F statistic and p value show that the null hypothesis can be rejected. The Monte-Carlo test indicates it is significant.

## 3 Results

### 3.1 Effect of different Si sources on phytolith content

**Figure 2:**
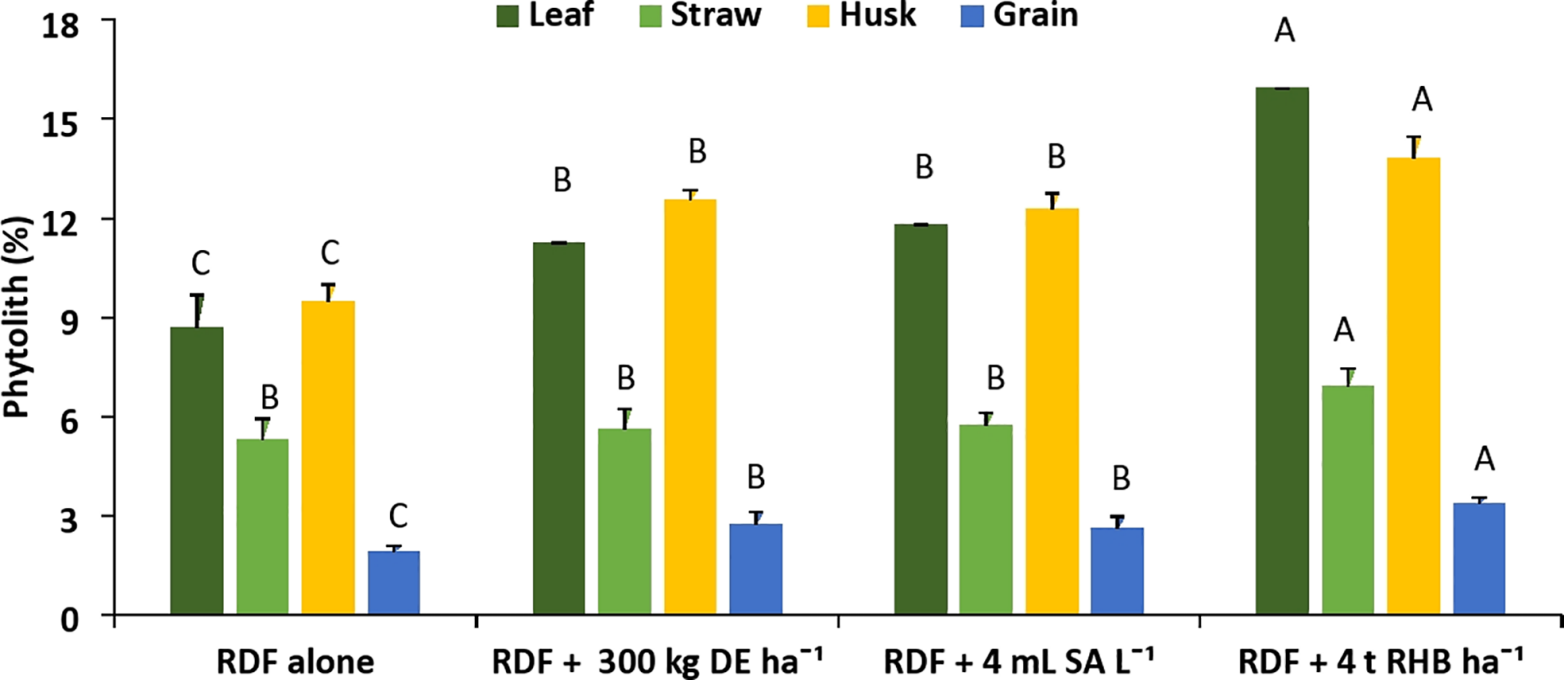
Effect of different sources of Si on phytolith content in leaf, straw, husk and grain of aerobic rice. (Capped bars at the surface of vertical bars represents the standard deviation, n = 4. Means ±SD with different letters in the same column indicate significant differences among the treatments tested (Tukey, p ≤ 0.05)DE, Diatomaceous Earth; SA, Silicic Acid; RHB, Rice Husk Biochar).

The content of phytolith significantly increased compared to the control (RDF alone) with application of different Si sources (**Figure 2**). The different Si sources increased the phytolith content of each rice organ and follow the trend: husk > leaf > straw > grain. The average phytolith content in all the treatments varied significantly from 1.83 to 13.80% among different rice organs. The content of phytolith in leaf (8.45 ± 0.59%) and straw (6.91 ± 0.55%) was significantly (*p<* 0.05) higher than that of the control. Among the treatments, application of 4 t RHB ha^-1^ had the highest phytolith content (8.45 ± 0.59%) in leaf, followed by 4mL SA L^-1^ (8.02 ± 0.64%) and 300 kg DE ha^-1^ (7.45 ± 0.34%). Phytolith content in straw was recorded to be higher with the treatment receiving 4 t RHB ha^-1^ (6.91 ± 0.55%) and the remaining treatments were on par with each other. In the husk, a significantly higher phytolith content of 13.80 ± 0.67% was recorded with the application of 4 t RHB ha^-1^, whereas DE application at 300 kg ha^-1^ (12.55 ± 0.30%) and SA application at 4 mL L^-1^ (12.28 ± 0.48%) produced comparable results. Phytolith content in grain follows the same trend with treatment receiving 4 t RHB ha^-1^ recording significantly higher phytolith (3.41 ± 0.16%) followed by 300 kg DE ha^-1^ (2.73 ± 0.39%) and 4 mL SA L^-1^ (2.61 ± 0.37%).

### 3.2 Effect of different Si sources on C content in phytolith and PhytOC content

**Figure 3:**
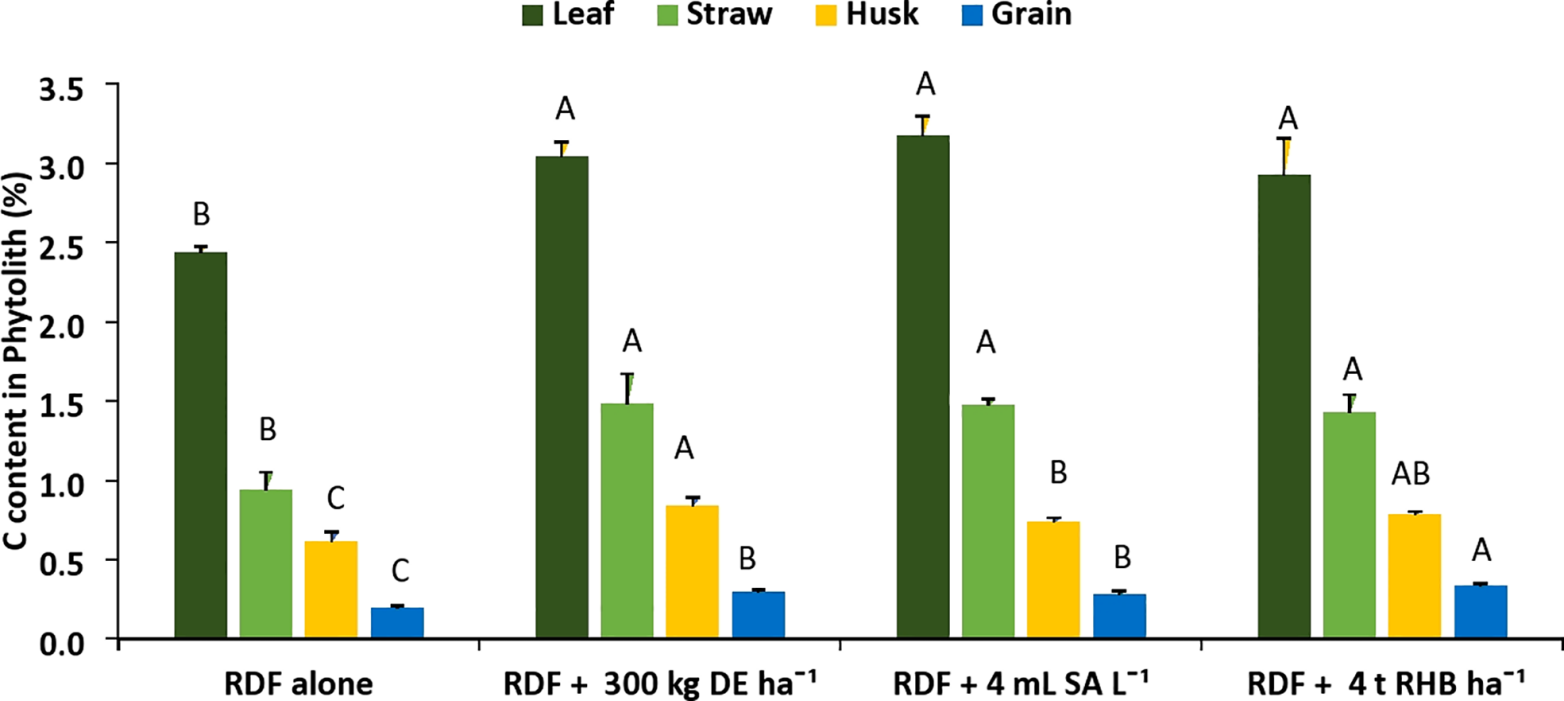
Effect of different sources of Si on C content in phytolith content in leaf, straw, husk and grain of aerobic rice. (Capped bars at the surface of vertical bars represents the standard deviation, n = 4. Means ±SD with different letters in the same column indicate significant differences among the treatments tested (Tukey, p ≤ 0.05). DE, Diatomaceous Earth; SA, Silicic Acid; RHB, Rice Husk Biochar).

**Figure 4:**
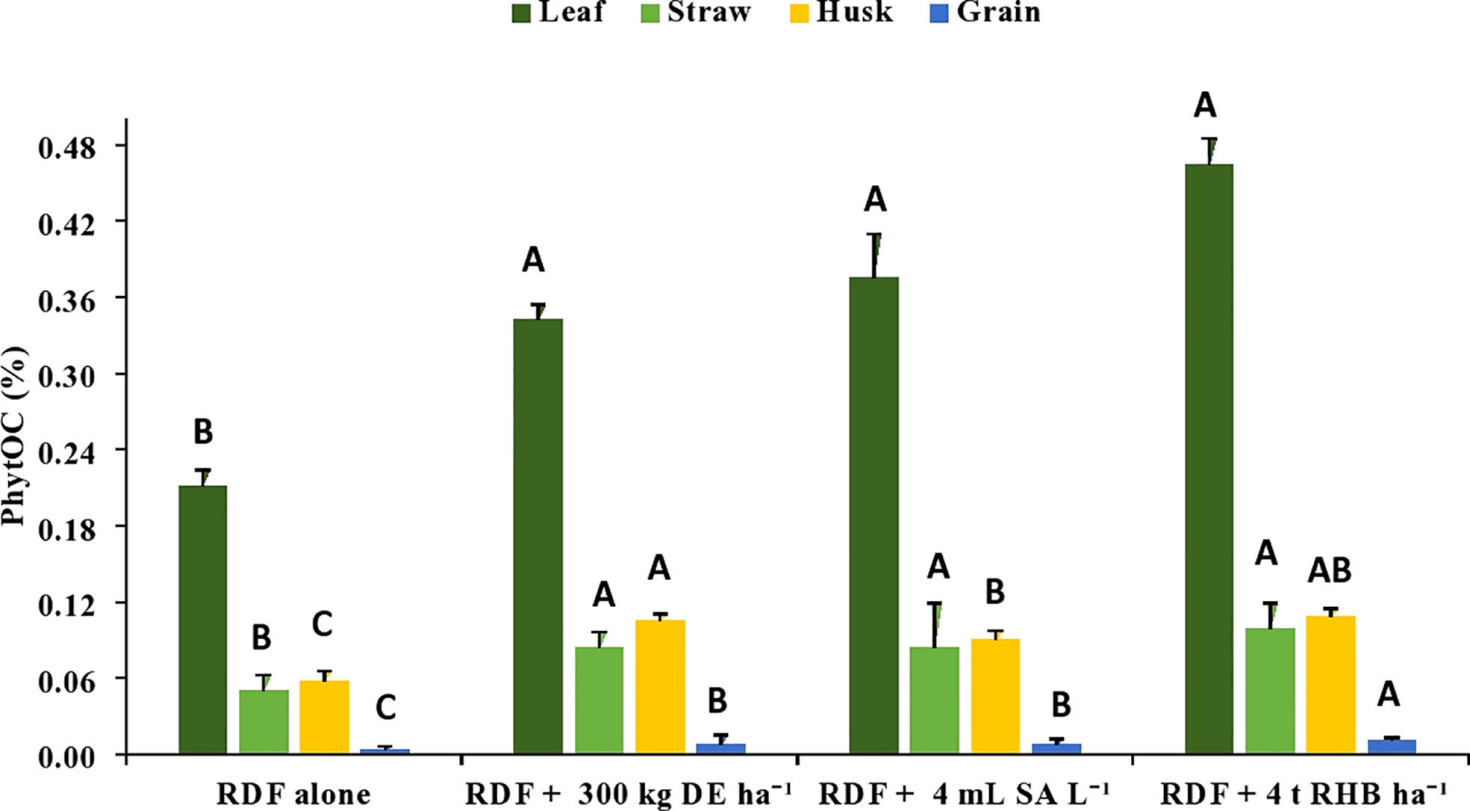
Effect of different sources of Si on phytOC in leaf, straw, husk and grain during of aerobic rice. (Capped bars at the surface of vertical bars represents the standard deviation, n = 4. Means ±SD with different letters in the same column indicate significant differences among the treatments tested (Tukey, p≤ 0.05). DE, Diatomaceous Earth; SA, Silicic Acid; RHB, Rice Husk Biochar; PhytOC, Phytolith Occluded Carbon).

There was a significant increase in the C content in phytolith (%) and PhytOC (%) with the application of different Si sources (**Figures 3**, **4**). However, it does not follow any trend within different rice organs and different sources of Si. The C content in the phytolith and PhytOC content in different rice organs ranged from 0.19 to 3.04% and 0.004 to 0.46%, respectively. Different Si sources significantly increased the C content in Phytolith (%) in leaf over control (RDF) but there was no significant difference among the treatments. Treatment receiving 300 kg DE ha^-1^ recorded higher C content in straw phytolith (1.48 ± 0.45%) and husk phytolith (0.84 ± 0.05%) (**Figure 3**). The C content in grain phytolith was found to be higher in the treatment receiving 4 t RHB ha^-1^ (0.33 ± 0.01%) and lower in the control (0.19 ± 0.025%) (**Figure 4**). Application of 4 t RHB ha^-1^ had significantly higher PhytOC content in leaf (0.46 ± 0.02%) and grain (0.011 ± 0.001%), while the control (RDF alone) recorded the lowest (**Figure 4**). Whereas PhytOC content in straw and husk did not differ significantly with the application of different sources of Si (**Figure 4**).

### 3.3 Effect of different Si sources on physicochemical properties of soil

There was no significant difference in pH and EC among the treatments (**Table 2**). The average OC content in post-harvest soil ranged from 5.27 ± 0.66 to 6.79 ± 0.63 g kg^-1^ (4 t RHB ha^-1 ^= 4 mL SA L^-1^ > 300 kg DE ha^-1^> Control (RDF)) recorded significantly (p<0.05) higher organic carbon (OC) (**Table 2**). The available N content in soil varied from 145.60 ± 7.92 to 198.80 ± 5.60 kg ha^-1^ and showed the trend: 300 kg DE ha^-1^ > 4 mL SA L^-1^ > 4 t RHB ha^-1^ > Control (RDF) (**Table 2**). Available P_2_O_5_ content varied significantly between 190.24 ± 8.79 to 276.10 ± 14.79 kg ha^-1^ and followed the trend: 300 kg DE ha^-1 ^= 4 t RHB ha^-1^ > 4 mL SA L^-1^ > Control (RDF) (**Table 2**). The average available K_2_O varied from 381.33 ± 10.48 to 414.03 ± 10.27 kg ha^-1^ (4 t RHB ha^-1 ^= 4 mL SA L^-1^> 300 kg DE ha^-1^> Control (RDF)) (**Table 2**).

**Table 2:** Effect of different sources of Si on pH, EC, OC, available N, P_2_O_5_ and K_2_O and different pools of Si in post-harvest soils of aerobic rice.

Treatments	pH	EC	OC	Available N	Available P_2_O_5_	Available K_2_O
		(dS m^-1^)	(g kg^-1^)	----------(kg ha^-1^)----------		
**T_1_:RDF alone**	8.21±0.03A	0.26±0.07A	5.27±0.66B	145.60±7.92C	190.24±8.79C	381.33±10.48B
**T_2_:RDF + DE @ 300 kg ha^-1^**	8.26±0.04A	0.35±0.04A	6.19±0.32AB	198.80±5.60A	276.10±14.79A	398.06±9.19AB
**T_3_: RDF + SA @ 4 mL L^-1^**	8.24±0.09A	0.26±0.04A	6.67±0.35A	193.20±3.23AB	221.46±12.93B	413.11±15.00A
**T_4_: RDF + RHB @ 4 t ha^-1^**	8.27±0.l0A	0.25±0.02A	6.79±0.63A	180.60±9.56B	266.16±11.97A	414.03±10.27A
**S.Em±**	**0.04**	**0.03**	**0.25**	**3.50**	**6.16**	**5.75**
**C. D. @ 5%**	**NS**	**NS**	**0.80**	**10.90**	**19.18**	**17.84**
**c.v. (%)**	**0.92**	**16.10**	**8.26**	**3.90**	**5.16**	**2.85**

±Values indicated standard deviation Means ±SD with different letters in the same column indicate significant differences among the treatments tested (Tukey, p ≤ 0.05).

DE, diatomaceous earth; SA, silicic acid; RHB, rice husk biochar.

For non-significant (NS) data Critical difference (CD) value is not calculated. It is reported as NS.

### 3.4 Effect of different Si sources on readily soluble Si pools

**Figure 5:**
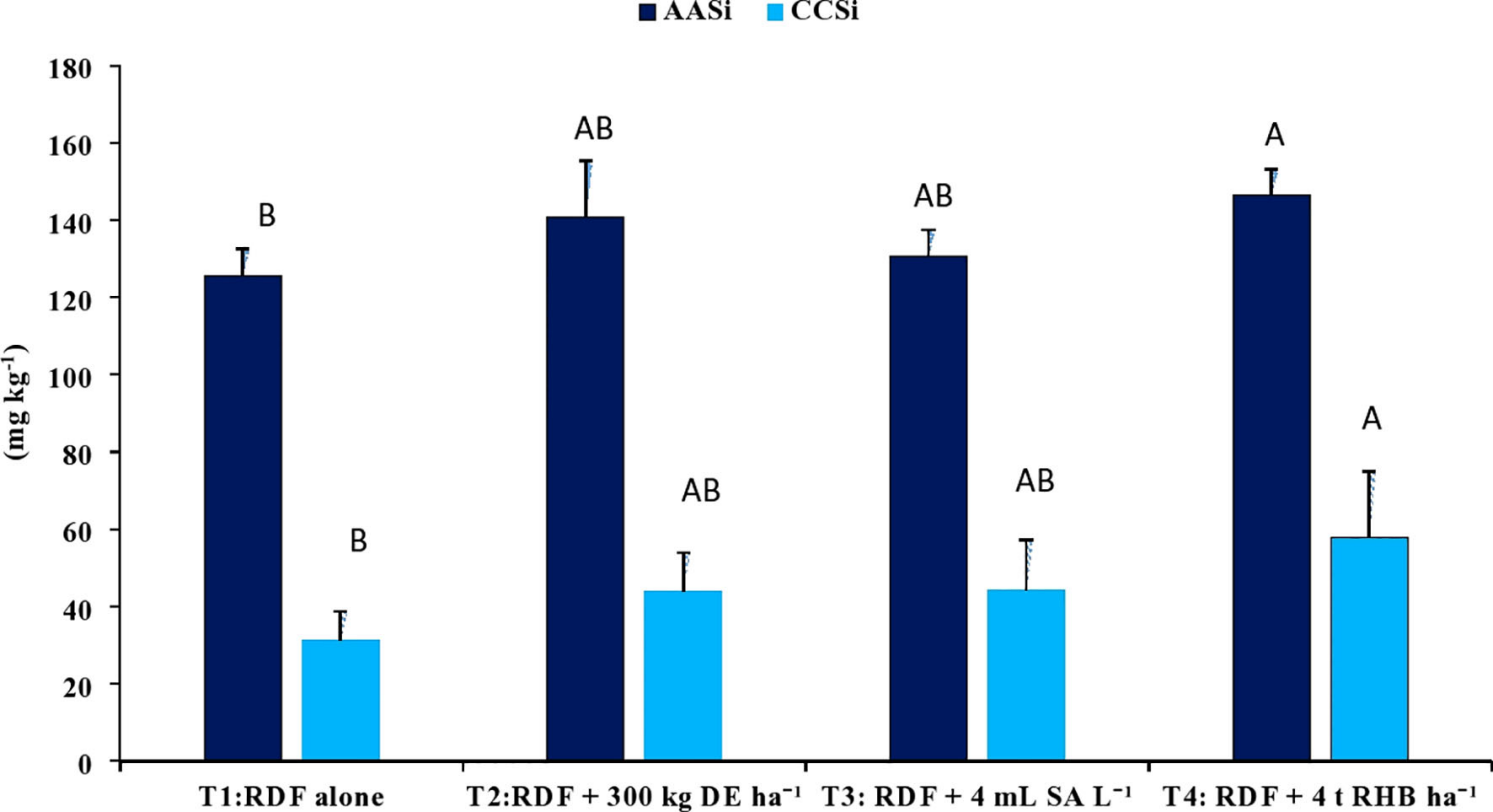
Effect of different sources of Si on CCSi and AASi content in post harvest soils of aerobic rice. (Capped bars at the surface of vertical bars represents the standard deviation, n = 4. Means ±SD with different letters in the same column indicate significant differences among the treatments tested (Tukey, p < 0.05). DE, Diatomaceous Earth; SA, Silicic Acid; RHB, Rice Husk Biochar; CCSi, 0.01 M Calcuim chloride extractable Si; AASI, 0.5 M Acetic acid extractable Si).

**Figure 6:**
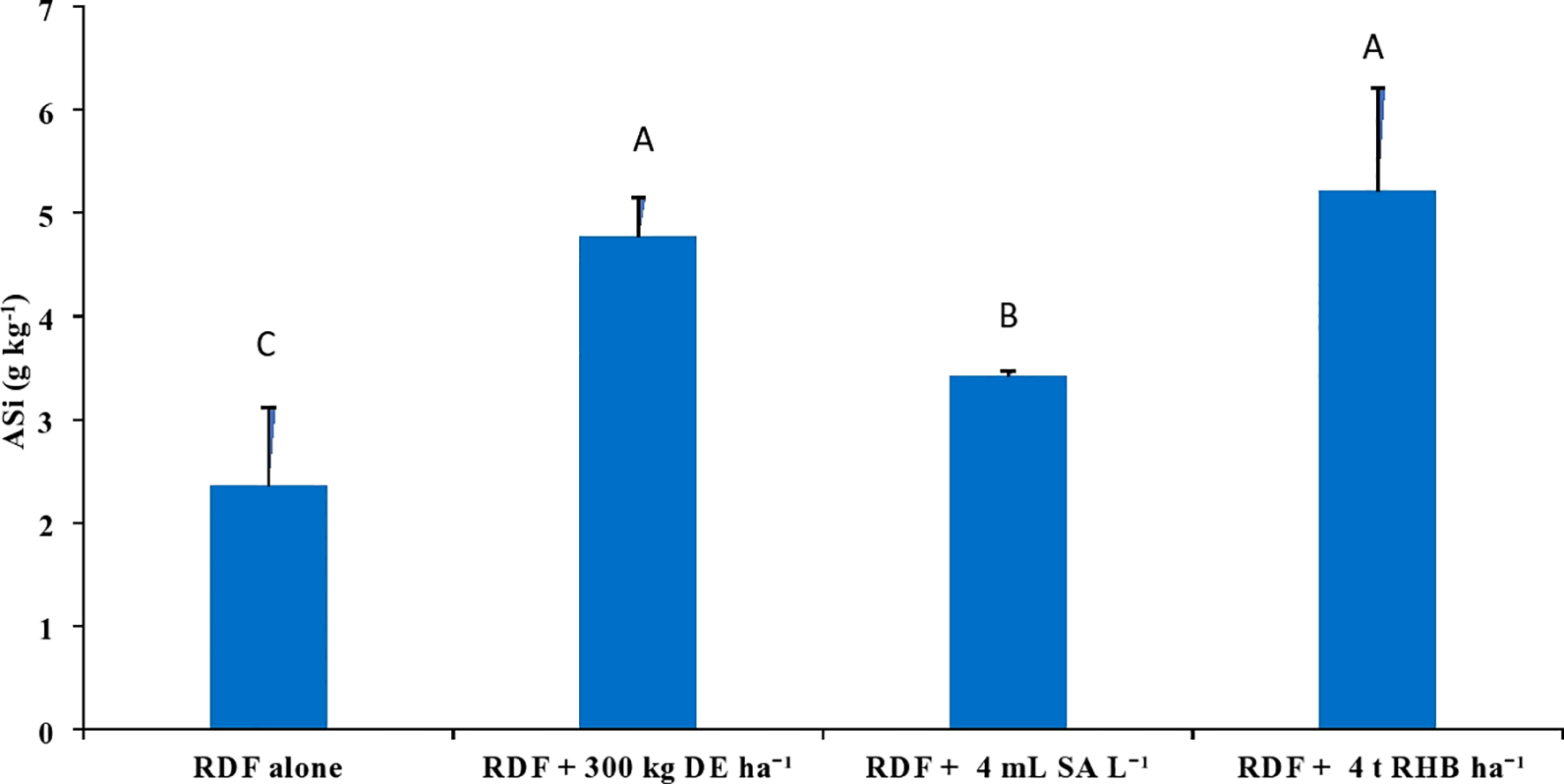
Effect of different sources of Si on ASi con tent in post harvest soils of aerobic rice. (Capped bars at the surface of vertical bars represents the standard deviation, n = 4. Means ±SD with different letters in the same column indicate significant differences among the treatments tested (Tukey, p≤ 0.05). DE, Diatomaceous Earth; SA, Silicic Acid; RHB, Rice Husk Biochar; ASi, Amorphous Si).

With the application of different silicon sources, the content of 0.5 M acetic acid extractable Si (AASi) and 0.01 M calcium chloride extractable Si (CCSi) in the soil increased significantly (p<0.05). Treatment receiving 4 t RHB ha^-1^ resulted in significantly higher AASi (146.56 ± 6.52 mg kg^-1^), CCSi (57.99 ± 17.01 mg kg^-1^) and ASi content (5.21 ± 1.00 g kg^-1^) values, while RDF resulted in the lowest (**Figures 5**, **6**). Application of 300 kg DE ha^-1^ and 4 mL SA L^-1^ produced comparable results.

### 3.5 Redundancy analysis

**Figure 7:**
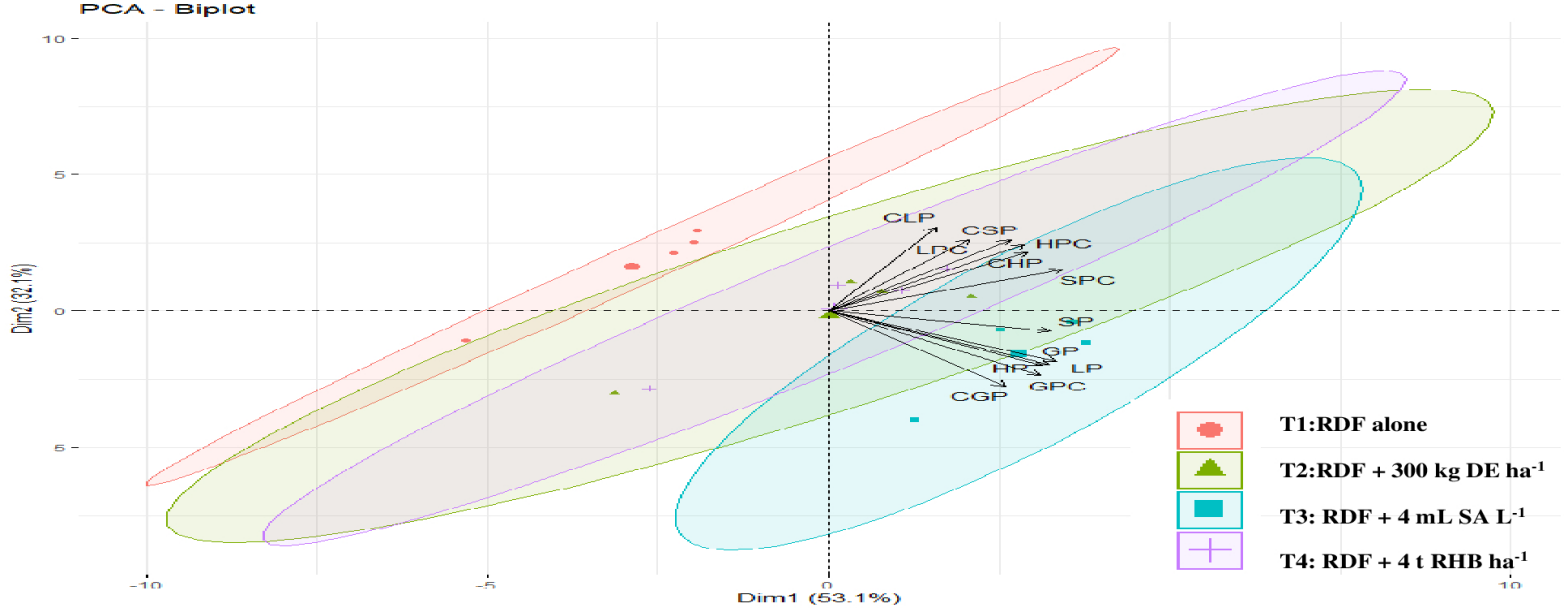
Principal component analysis (PCA) of plant phytolith and phytOC parameter of different rice organs in response to different sources of Si. (DE, Diatomaceous Earth; SA, Silicic Acid; RHB, Rice Husk Biochar; LP, Leaf phytolith; SP, Straw phytolith; HP, Husk phytolith; GP, grain phytolith; CLP, carbon content in leaf phytolith, CSP, carbon content in straw phytolith; CHP, carbon content in husk phytolith; CGP, carbon content in grain phytolith; LPC, leaf phytOC; SPC, straw phytOC; HPC, husk phytOC; GPC, grain phytOC).

**Figure 8:**
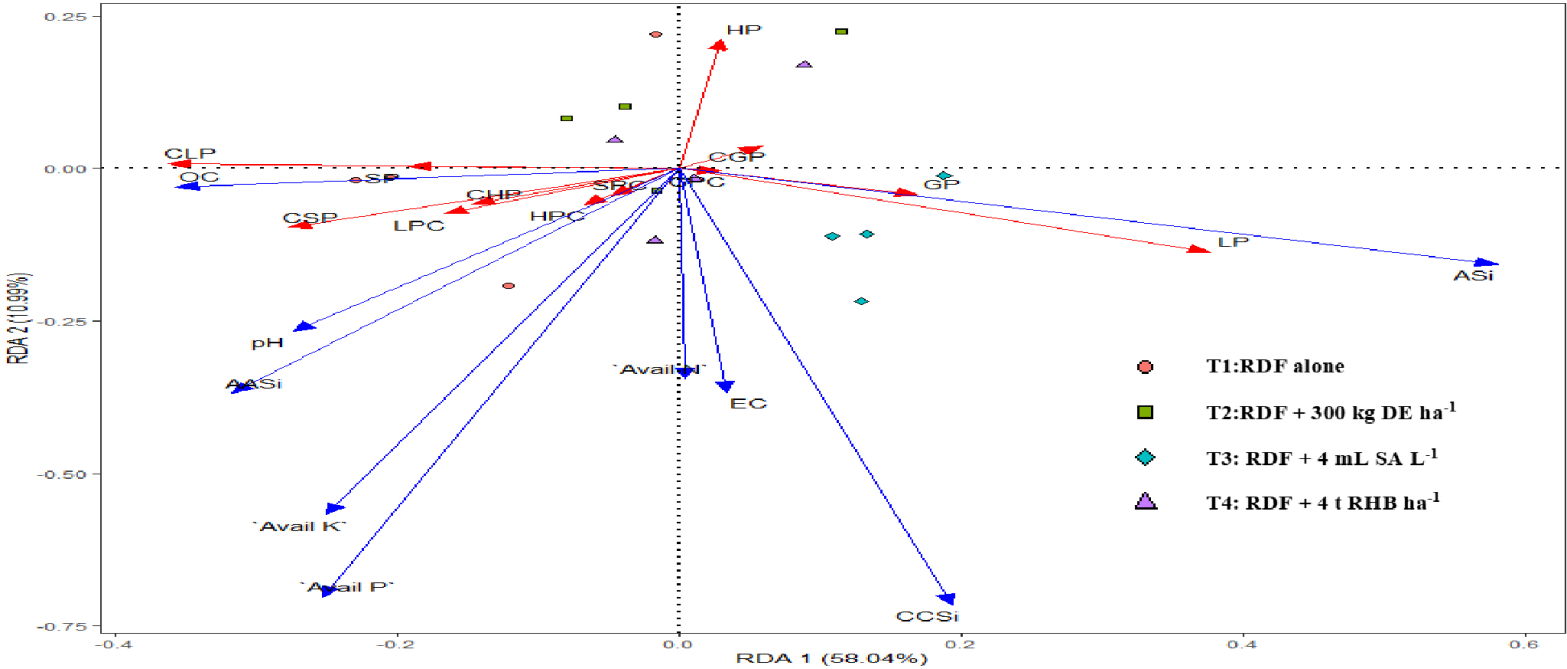
Redundancy analysis (RDA) of physicochemical properties of soil in response to different sources of Si (DE, Diatomaceous Earth; SA, Silicic Acid; RHB, Rice Husk Biochar; OC, Soil Organic Carbon; Avail N, Available Nitrogen; Avail P_2_O_5_, Available Phosphorus; Avail K_2_O, Available Potassium; CCSi, 0.01 M CaCl_2_ extractable Si; AASi, 0.5 M Acetic Acid extractable Si and ASi - Amorphous SILP, Leaf phytolith; SP, Straw phytolith; HP, Husk phytolith; GP, grain phytolith; CLP, carbon content in leaf phytolith; CSP, carbon content in straw phytolith; CHP, carbon content in husk phytolith; CGP, carbon content in grain phytolith; LPC, leaf phytOC; SPC, straw phytOC; HPC, husk phytOC; GPC, grain phytOC).

In this study, we conducted a redundancy analysis to explore the relationships between phytolith and PhytOC content in plants and readily available soil Si pools and soil properties, and how those relationships are affected by different Si sources. Plant parameters (phytolith, C content in phytolith and PhytOC) were considered response variables. Whereas, soil parameters (pH, EC, OC, available N, P, K, CCSi, AASi, and ASi) were explanatory variables. RDA is the multivariate (meaning multi-response) technique analogue of regression. The technique employs a combination of linear regression and principal component analysis (PCA). A PCA analysis was performed on the response variables to generate a set of principal component vectors for the RDA analysis. RDA uses a mix of linear regression and principal component analysis (PCA). To conduct the RDA analysis, a PCA analysis was performed on the response variables to get a set of principal component vectors. Response variables were quantified using principal component analysis and classified as strong, moderate, or weak based on loading values greater than 0.75. Two factors (Principal Components, PC) were extracted that explained 87.3% of the total observed variation in plant phytolith and PhytOC content Eigenvalues greater than 1.0. (**Table 3**). PC1 explains 62.4% of the variance and was correlated (Factor loading > 0.75) with the phytolith content in the leaf, straw, husk and grain (**Table 3**, **Figure 7**). PC2 explains the 24.9% variance with the strong loading of the C content and PhytOC content in the leaf, straw, husk, and grain. RDA1 and RDA2 total explained proportions were 58.03 and 10.99%, respectively (**Figure 8**). The model’s significance was determined using Monte Carlo permutation tests (999 permutations). The RDA revealed that the concentrations of OC (F = 5.26, p = 0.06), available N (F = 12.36, p = 0.004***), available K (F = 6.91, p = 0.02*), CCSi (F = 8.16, p = 0.01*), and ASi (F = 44.96, p = 0.001***) were the most important factors influencing the phytolith and PhytOC parameters, as evidenced by significant correlations (**Table 4**).

**Table 3:** Rotated component matrix of plant phytolith, C content in phytolith and PhytOC content in different rice organs.

Variables	Components	
	1	2
**LP**	0.942	0.130
**SP**	0.754	0.376
**HP**	0.925	0.106
**GP**	0.945	0.171
**HPC**	0.170	0.931
**SPC**	0.431	0.839
**LPC**	-0.013	0.837
**GPC**	0.023	0.974
**CHP**	0.228	0.880
**CSP**	0.107	0.932
**CLP**	-0.181	0.849
**CGP**	-0.127	0.940

Extraction Method: Principal Component Analysis

Rotation Method: Varimax with Kaiser Normalization.

LP, Leaf phytolith; SP, Straw phytolith; HP, Husk phytolith; GP, grain phytolith ; CLP, carbon content in leaf phytolith; CSP, carbon content in straw phytolith; CHP, carbon content in husk phytolith; CGP, carbon content in grain phytolith; LPC, leaf PhytOC; SPC, straw PhytOC; HPC, husk PhytOC; GPC, grain PhytOC.

**Table 4:** Monte Carlo permutation tests (999 permutations) of the effect of physicochemical properties of soil on plant phytolith, C content in phytolith and PhytOC content.

Parameter	F. model	R2	P
pH	5.26	0.040	0.06
EC	-0.26	-0.001	-1.00
OC	8.16	0.062	0.01*
Avail. N	12.36	0.094	0.004***
Avail. P_2_O_5_	0.74	0.006	0.48
Avail. K_2_O	6.91	0.053	0.02*
CCSi	43.20	0.330	0.001 ***
AASi	3.56	0.027	0.07
Asi	44.97	0.34351	0.001 ***

*** Significant at ≤0.001; ** Significant at p ≤0.01; * Significant at p≤0.05.

## 4 Discussion

### 4.1 Plant phytolith and PhytOC content

In our study, the phytolith content, C content in phytolith and PhytOC content in different rice organs increased with the application of different sources of Si (**Figures 2**–**4**). Variation in silica deposition in different rice parts is caused by passive transport i.e., organs with higher transpiration rates accumulate more Si (Kumar et al., 2017). On the other hand, Yamaji et al. (2015) found that despite having a lower transpiration rate, the rice husk accumulated more silica due to the presence of two developed vascular bundles below the panicle. The higher Si flow in the husk gives the hard outer coating of grain which protects grain during development. Our findings are consistent with the findings of Gallo et al. (1974), who studied rice, oat, rye, and wheat and reported that the seed coat accumulates the most silica and the grain accumulates the least. A significant positive correlation among phytolith, C content in phytolith and PhytOC have been reported by various researchers (Anjum and Prakash, 2021; Rehman et al., 2023). PhytOC content in plants depends on the phytolith content and the C encapsulation potential of the phytolith during the formation (Anjum and Prakash, 2021). Among the different sources of Si, biochar performed better because plant-derived phytoliths are concentrated in biochar (Li et al., 2020b). Pyrolysis of Si-rich crop residue enhances solubility of phytoliths and their ability to release PASi (Li et al., 2020b), Si uptake and crop biomass (Li et al., 2019b; Li et al., 2019c). Increased PASi with Si fertilizer and biochar application indirectly increased leaf C content in phytolith, improving PhytOC accumulation of Moso bamboo, particularly in treatment receiving biochar (Huang et al., 2020). Guo et al. (2015) found that basaltic powder (BP) increased the phytolith and PhytOC due to increased rice silicon uptake, which accelerated the chemical weathering of primary silicate minerals in basalt and thus the release of dissolved silicon. Li et al. (2020a) found that applying Si-P fertilizer in Si-deficient soils has no discernible effect on phytoliths and phytOC. Sun et al. (2019) conducted a pot experiment in Si-enriched and Si-deficient soils and reported that slag-based Si fertilizer increased phytolith and PhytOC concentration under both conditions. Thus, the external supply of Si through fertilizer and biochar has an impact on phytolith accumulation and PhytOC concentration.

### 4.2 Changes in physicochemical properties of soil

Application of RHB and Si fertilizers had no significant effect on soil pH and EC over control (RDF) (**Table 3**). Although there was an increase in the soil pH compared to initial values, EC had no definite trend. The initial pH of the experimental soil in the year 2015 was 7.10 but due to continuous monocropping, the pH of the experimental field has increased to 8.2. Xiang et al. (2009) reported that an increase in soil pH from 6.4 to 7.1 after 12 seasons of aerobic cultivation as the soil reduction-driven moderation or amelioration in soil pH observed in submerged rice soils is not observed in aerobic soils. Similar results with the application of DE (Sandhya and Prakash, 2018), calcium silicate, rice, hull and rice hull ash (Sandhya and Prakash, 2017) and biochar (Wu et al., 2021) have been reported in neutral or alkaline soil. There was a significant increase in the SOC content in the treatment receiving 4 t RHB ha^-1^ over control by ~ 29% (**Table 3**). Improvement in available nitrogen (N), phosphorus (P) and potassium (K) was found with the application of different sources of Si (**Table 3**). Similar results were reported by Sandhya and Prakash (2017). Increase in available N status in soil caused by N and Si fertilization could be attributed to high adsorption capacity of Si and increased microbial activity, which accelerated the mineralization process during the crop growth period, resulting in a high N accumulation in Si-treated soils. Similarly, Yogendra et al. (2017) found that using CaSiO_3_ increased N-use efficiency in aerobic rice. In the current investigation, treatment receiving biochar increased SOC significantly, but available N did not follow the same trend, which could be attributed to a high C: N ratio in crop residue-derived biochar (RHB). Similar results were reported by Han et al. (2020) where biochar amendment led to a significant decrease in dissolved organic nitrogen (DON) concentration in both low-fertility and high-fertility soil. Also, slight decrease in the available N several folds from initial year of experiment 2015 (340.80 kg ha^-1^ to summer 2019 (145 to 180 kg ha^-1^). Because of higher nitrogen losses in form of nitrous oxide (N_2_O), through coupled nitrification-denitrification processes. Improvement in the available phosphorus (P) with the application of Si over control can directly increase the available P by reducing Fe-P retention capacity under higher PASi which converts slightly soluble phosphate into plant-available forms (Schaller et al., 2019). Increase in available K_2_O content of the soil with the application of Si could be due to the utilization of native K_2_O with increasing levels of silicon which resulted in the building up of higher soil K_2_O status. The increased available K with Si application could be ascribed to the alterations in crystal structures of clay lattices which might have been related to a reduction in K fixation thus releasing potassium. The application of biochar significantly increased the available K_2_O due to a large amount of K absorbed on the surface of biochar are bioavailable and increased activity of K-dissolving bacteria (Xia et al., 2022). The contents of available P and K were increased during the cultivation process (from the year 2015-2019). This was likely due to fertilizer addition during all cultivation seasons. Together, these results suggest that application of Si fertilizer and RHB improves the soil physicochemical properties.

### 4.3 Changes in readily soluble Si pools

Addition of phytolith-rich biochars in cultivated soils promotes the release of PASi in the soil-plant system (Li and Delvaux, 2019a), therefore, RHB application resulted in higher CCSi and AASi (**Figure 5**). The application of the biochar produced at a lower pyrolytic temperature (700°C) phytolith exposure is more, as a result, higher dissolution of phytolith (Xiao et al., 2014; Li and Delvaux, 2019a). According to the Si dissolution kinetics, high Si biochar could be a novel slow-release source of biologically available Si in low Si agricultural soils (Xiao et al., 2014). The increased available Si was anticipated with the application of DE as the added DE might have contributed to the release of soluble Si (Sandhya and Prakash, 2018; Sandhya and Prakash, 2019).

The ASi content in this study was analyzed as per Majumdar and Prakash (2020b), which was optimized for the estimation of ASi content in tropical soils. In this study, the soil samples were analyzed in duplicate for each treatment per replication for 3 to 6 h. A regression model was prepared from 3 to 6 h has significant variables and produces a higher value of the coefficient of determination (R^2^) (**Table S1**). ASi content in this study ranged from 2.36 to 5.21 g kg^-1^ (**Figure 6**), which was found to be consistent with the previous studies (Guntzer et al., 2012; Nguyen et al., 2019; Majumdar and Prakash, 2020a). Because of the presence of abundant phytolith, the treatment receiving RHB had an empirically higher ASi content. Li et al. (2019c) and Li et al. (2020b) found that using Si (+) biochar resulted in a higher ASi in comparison with Si (-) biochar and wollastonite. Similarly, Majumdar and Prakash (2020b) reported a higher ASi content in the sugarcane soil profile than the rice soil due to the incorporation of burnt sugarcane trash into the soil during harvesting. ASi content in the control (RDF) was lower due to the continuous removal of rice straw from the field. Because, rice cultivation intensifies desilication as rice straw contains 86% of Si taken up by rice plants (Anjum and Prakash, 2021). Dissolution rate of ASi is several orders of magnitude higher than those of kaolinite or plagioclase at near-neutral pH (Riotte et al., 2017). Si isotopic signatures study conducted by Riotte et al. (2017) confirmed that the ASi plays an important source of Si in rice. Guntzer et al. (2012) reported that the soil ASi pool (0.5 mg g^-1^ to 4.3 mg g^-1^) in Broadbalk, Rothamsted (England) depleted with the export of wheat straw from 1883 to 1944, particularly in surface soil. Desplanques et al. (2006) estimated that irrigation waters bring more than one-third of the Si needed for rice. They conducted solute mass balance study in the rice field of Camargue (France) where DSi in irrigation water was low and rice straw was removed from the field. They hypothesized that ASi being the only source of dissolved silicon, will be exhausted in 5 years. Since our experiment was a 5-year-old intensive rice experiment plot (10 seasons) converted to the aerobic rice experiment for the past 2 years (4 seasons) the risk of ASi depletion in the soil is very high.

### 4.4 Plant phytolith, C content in phytolith, and PhytOC content in rice in relation to soil properties

PCA data showed that the plant phytolith and PhytOC parameters under investigation were divided into four major groups (**Figure 7**). According to the ordination of variables in the PCA diagram, variables in control (RDF) appeared to not correlate with phytolith and PhytOC content, whereas the external Si application appeared to have a positive correlation. Among the treatments, the phytolith and PhytOC parameters of the treatment receiving 300 kg DE ha^-1^ and 4 mL SA L^-1^ clustered together. In this study, the application of biochar increased the plant phytolith and PhytOC content over other Si fertilizer treatments. Whereas, Huang et al. (2020) found that biochar had no effect on PhytOC content in the Moso bamboo plantation but did influence leaf phytolith formation. Several studies have previously found that biochar improves phytolith concentration (Si uptake) in cotton (Li et al., 2018), wheat (Li et al., 2019c) and rice (Wang et al., 2019). However, the impact was caused by variations in vegetation type, source and rate of biochar and PASi (Li and Delvaux, 2019a; Wang et al., 2019).

Monte Carlo permutation tests revealed significant relationships between the concentrations of plant phytolith and PhytOC as well as SOC, available N, available K_2_O, CCSi and ASi indicating close association with each other (**Table 4**). **Figure 8** shows that the RDA ordination of variables revealed that the samples of treatments receiving 4 t RHB ha^-1^ have a stronger relationship with the CCSi and ASi. Huang et al. (2020) found that PASi concentration was positively correlated with plant phytolith parameters (F = 8.1, P = 0.001), particularly in Si fertilizer-amended soil subtropical bamboo in China. Tan et al. (2021) reported that soil nutrient availability significantly influenced the spatial variability of phytoliths and PhytOC concentration in rice. Since Si is the most abundant element in phytoliths, and PASi in soil has a strong correlation with straw phytolith concentration, soil nutrient availability may influence phytolith concentration by affecting plant-available Si concentration. There was a significant correlation among the SOC, phytolith and PhytOC content in different organs of rice. Similarly, Yang et al. (2020) reported a significant positive correlation between CCSi and ASi (soil phytolith) when phytolith-rich rice residue was applied. This could be due to the accumulation of soil organic matter, which when mineralized, releases phytoliths. As a result, SOC may affect the phytolith concentration by influencing soil Si availability. Contrary to our findings, Tan et al. (2021) and Ji et al. (2019) reported that SOC decreased with the increasing level of Si input. According to some studies, soil silica availability is significantly positively correlated with soil pH (Schaller et al., 2019; Majumdar and Prakash, 2021). As a result, soil pH may have a positive correlation with phytolith concentration, which is consistent with our findings. N, P, K, Si fertilization and biochar addition not only increase biomass production but also increase phytolith production and PhytOC sequestration (Song et al., 2017). This study on the effect of different Si sources (DE, SA and RHB) in rice is the first field study and a step forward from previous laboratory experiments (Guo et al., 2015; Sun et al., 2019; Li et al., 2020a). It has been previously reported that the phytolith and C content in the phytolith influences the plant PhytOC (Sun et al., 2019; Li et al., 2020a; Anjum and Prakash, 2021). The addition of different Si sources (Si fertilizers and RHB) significantly impacts the former, with increased soil CCSi and ASi as a result of Si fertilizer and RHB application.

## 5 Conclusions

This is the first study to assess the phytolith and PhytOC production in the intensively cultivated rice ecosystem as it is influenced by different Si sources. In response to our objective, we can conclude that continuous application of Si fertilizers and RHB significantly (i) increased the phytolith and PhytOC content, (ii) improved soil physicochemical properties, (iii) increased the readily soluble Si pools (CCSi, AASi, and ASi). However, the effect was more pronounced in treatment receiving RHB. Phytolith and PhytOC production is closely associated with the soil physicochemical properties and readily available Si pools. Crop management practices (straw management, N, P, K, and Si fertilizers and amendments) alter soil physicochemical properties, influencing phytolith and PhytOC accumulation in plants. The importance of Si fertilizers in sustaining crop productivity in rice-rice cropping systems and coupled Si and C cycles was demonstrated in this study. It also emphasizes the significance of the continuous application of phytolith-rich biochars like RHB as a cost-effective and sustainable alternative to increasing phytolith and PhytOC production in the rice ecosystem.

## Data availability statement

The raw data supporting the conclusions of this article will be made available by the authors, without undue reservation.

## Author contributions

MA and NP conceived the idea and collected the relevant literature. MA visualized the figures. MA and NP helped in writing the original draft. All authors carefully read, revised, and approved the article for submission.
