# Supplementary material for: Production of phytolith and PhytOC and distribution of extractable Si Pools in aerobic rice as influenced by different Si sources
Source: Front Plant Sci. 2023 Aug 24;14:1146416. doi: 10.3389/fpls.2023.1146416 (PMC10486906; doi:10.3389/fpls.2023.1146416)
Supplement: Supplementary file 1 [file Table_1.docx]

**Table S1 |** Corrected ASi (g kg^-1^) content in post-harvest soils of aerobic rice during *summer* 2019 from 3 to 6 h basis (Majumdar and Prakash, 2019)

| **Treatments** | **Regression**  **equation** | **Coefficient of**  **determination (R^2^)** | **Coefficient of correlation (r)** | ***p* values**  **two tailed** | **Corrected**  **ASi (%)** | **Corrected**  **ASi (g kg^-1^)** |
| --- | --- | --- | --- | --- | --- | --- |
| **T1:RDF alone** | y = 0.1359x + 0.2361 | 0.91 | 0.96 | 0.0009 | 0.2361 | 2.361 |
| **T2:RDF + 300 kg DE ha^-1^** | y = 0.2702x + 0.4852 | 0.94 | 0.97 | 0.0001 | 0.4852 | 4.852 |
| **T3: RDF + 4 mL SA L^-1^** | y = 0.1151x + 0.3448 | 0.95 | 0.98 | 0.0001 | 0.3448 | 3.448 |
| **T4: RDF + 4 t RHB ha^-1^** | y = 0.2634x + 0.5212 | 0.89 | 0.94 | 0.0005 | 0.5212 | 5.212 |

x is the distance of a particular point while estimating ASi content from the y - axis on a graph in the Cartesian coordinate system

**Corrected ASi (%) is calculated at p ≤ 0.05

Abbreviation: DE - Diatomaceous Earth, SA - Silicic Acid, RHB - Rice Husk Biochar and ASi - Amorphous Silicon
